# Reactive case-detection of malaria in Pailin Province, Western Cambodia: lessons from a year-long evaluation in a pre-elimination setting

**DOI:** 10.1186/s12936-016-1191-z

**Published:** 2016-03-01

**Authors:** John Hustedt, Sara E. Canavati, Chandary Rang, Ruth A. Ashton, Nimol Khim, Laura Berne, Saorin Kim, Siv Sovannaroth, Po Ly, Didier Ménard, Jonathan Cox, Sylvia Meek, Arantxa Roca-Feltrer

**Affiliations:** Malaria Consortium Cambodia, Phnom Penh Office, House #91, St. 95, Boeung Trabek, Chamcar Morn, Phnom Penh, Cambodia; Faculty of Tropical Medicine, Mahidol University, 420/6 Ratchawithi Road, Ratchathewi, Bangkok, 10400 Thailand; Malaria Consortium Cambodia, 113 (6th floor of Parkway Square), Mao Tse Toung Blvd. Chamcar Morn, Phnom Penh, Cambodia; Institut Pasteur in Cambodia, 5, Blvd Monivong, Phnom Penh, Cambodia; The National Center for Parasitology, Entomology and Malaria Control, Ministry of Health, Corner street 92, Trapaing Svay village, Sankat Phnom Penh Thmey, Khan Sensok, Phnom Penh, Cambodia; Department of Disease Control, Faculty of Infectious and Tropical Diseases, London School of Hygiene and Tropical Medicine, London, UK; Malaria Consortium, Development House, 56-64 Leonard Street, London, EC2A 4LT UK; Faculty of Tropical Medicine, Malaria Consortium Asia, Mahidol University, Room 805, 420/6 Rajavidhi Road, Bangkok, 10400 Thailand

## Abstract

**Background:**

As momentum towards malaria elimination grows, strategies are being developed for scale-up in elimination settings. One prominent strategy, reactive case detection (RACD), involves screening and treating individuals living in close proximity to passively detected, or “index” cases. This study aims to use RACD to quantify *Plasmodium* parasitaemia in households of index cases, and identify risk factors for infection; these data could inform reactive screening approaches and identify target risk groups.

**Methods:**

This study was conducted in the Western Cambodian province of Pailin between May 2013 and March 2014 among 440 households. Index participants/index cases (n = 270) and surrounding households (n = 110) were screened for *Plasmodium* infection with rapid diagnostic tests (RDT), microscopy and real-time polymerase chain reaction (PCR). Participants were interviewed to identify risk factors. A comparison group of 60 randomly-selected households was also screened, to compare infection levels of RACD and non-RACD households. In order to identify potential risk factors that would inform screening approaches and identify risk groups, multivariate logistic regression models were applied.

**Results:**

Nine infections were identified in households of index cases (RACD approach) through RDT screening of 1898 individuals (seven *Plasmodium vivax*, two *Plasmodium falciparum*); seven were afebrile. Seventeen infections were identified through PCR screening of 1596 individuals (15 *P. vivax, and* 22 % *P. falciparum/P. vivax* mixed infections). In the control group, 25 *P. falciparum* infections were identified through PCR screening of 237 individuals, and no *P. vivax* was found. *Plasmodium falciparum* infection was associated with fever (p = 0.013), being a member of a control household (p ≤ 0.001), having a history of malaria infection (p = 0.041), and sleeping without a mosquito net (p = 0.011). Significant predictors of *P. vivax* infection, as diagnosed by PCR, were fever (p = 0.058, borderline significant) and history of malaria infection (p ≤ 0.001).

**Conclusion:**

This study found that RACD identified very few secondary infections when targeting index and neighbouring households for screening. The results suggest RACD is not appropriate, where exposure to malaria occurs away from the community, and there is a high level of treatment-seeking from the private sector. Piloting RACD in a range of transmission settings would help to identify the ideal environment for feasible and effective reactive screening methods.

## Background

Due to increased international funding, political will, and a new generation of malaria diagnostic and treatment tools, the target to achieve malaria elimination is growing in momentum [1–3]. As of 2014, 34 of the 99 malaria-endemic countries have adopted strategies to become malaria-free within the next two decades [4, 5]. Their strategies largely involve shifting the focus from early diagnosis and treatment of febrile malaria cases, to active surveillance to facilitate early detection and treatment of every individual infection, including those who are afebrile [6]. In Cambodia, there has been a marked decrease of 81 % in annual cases due to *Plasmodium falciparum* since 2009 [7].

Although active case detection (ACD) is considered an essential element of malaria elimination [8–13] there is a lack of consensus about when this approach should be used and what specific methods are most effective [8, 14–16]. The World Health Organization (WHO) has defined ACD as “the operation carried out by surveillance agents who visit every locality in a defined area at regular intervals (usually monthly during the transmission season), in order to enquire for fever cases through individual house visits, and to test for malaria (and treat if positive) each suspected person so discovered” [17]. Reactive case detection (RACD) is one strategy commonly described or promoted for scale-up in elimination settings. RACD is usually defined as restricting ACD to individuals living in close proximity (household members and neighbours) to cases detected through the passive case detection (PCD) system [13, 15]. An earlier study had indicated that even when overall malaria transmission declines, zones of relatively high malaria risk can persist [18]. Case-finding through RACD essentially takes advantage of the spatial and temporal clustering of malaria infections associated with transmission hotspots [18–20].

Unlike PCD, RACD is also potentially an effective strategy for detecting afebrile malaria infections, which also tend to cluster in low-transmission settings [9]. However, the ability of RACD to detect afebrile infections depends on the sensitivity of the diagnostic test being used. It is likely that standard field diagnostic methods (RDT and microscopy) will fail to detect a substantial proportion of low-density parasitaemia [9–14, 21, 22] and it has been argued that more sensitive DNA-based detection methods, such as PCR or loop-mediated isothermal amplification (LAMP), are required [9–14, 21–26] to better detect clustering of asymptomatic malaria infections. The proportion of individuals with parasitaemia in index households was compared to surrounding and randomly-selected control households in order to explore how many additional cases could be detected by screening non-index households.

The objectives of the study presented here are to quantify *Plasmodium* parasitaemia in households of index cases detected by PCD in a low malaria transmission setting, and to evaluate potential risk factors for infection that will inform reactive screening approaches and identify high-risk groups.

## Methods

### Study site

Pailin province is located in the northwest of Cambodia. This area has repeatedly been the epicentre of emerging resistance of *P. falciparum* to anti-malarial drugs since the 1960s [24, 27], and was the first location at which *P. falciparum* resistance to artemisinin-based treatments was first identified [28]. In recent years, this area has been the focus of numerous intensive control programmes aimed at containing artemisinin resistance [29]. The number of malaria cases has decreased substantially in Pailin province within the previous ten years, from 113,855 cases in 2004 to 56,271 cases in 2014 (Malaria Information System, 2015).

Pailin has six health centres, staffed with a nurse and a midwife who can test, treat, and track malaria cases. In addition, Pailin has also a malaria alert system delivered through a network of village malaria workers (VMWs). Each of the 114 villages has two VMWs who are trained to diagnose malaria infections using rapid diagnostic tests (RDT), and to provide treatment according to national guidelines. VMWs routinely collect blood films and filter paper blood spots and send a short message service (SMS) to alert the public health facility and the national malaria programme when a new malaria case is identified [30]. At the initial time of this study, the first-line treatment in Pailin was atovaquone-proguanil (AP, Malarone, GlaxoSmithKline) and then shifted to dihydroartemisinin–piperaquine (DHA–PQP) towards the middle of the study until the end. Those who do not seek treatment at the public health facilities are not included in the malaria alert system and therefore not included in our study.

### Study design

RACD was implemented between May 2013 and March 2014 in 114 villages in Pailin in order to quantify the reservoir of afebrile *Plasmodium* parasitaemia in households of “index” cases. Index cases were defined as those testing positive for malaria through PCD at a health facility or through a VMW. A team comprised of health centre staff or VMWs (based on catchment areas) and Malaria Consortium staff was mobilized to follow up index cases at their homes within 3 days of the case being detected. Within the household of the index case, all other residents were invited to participate in the study. In addition, for every 15th index case identified, the five nearest households to the index case household were also invited to participate. For every 30th index case, the ten nearest households were invited to participate.

In addition, 60 randomly-selected control households were also screened. The randomly-selected households were stratified by case numbers; 30 households were selected from villages with more than five confirmed *Plasmodium* infections reported in MIS data during the previous year (“high incidence” control households) and 30 households were selected from villages with five or fewer confirmed infections during the previous year (“low incidence” control households). The number of cases, rather than incidence, was chosen for purposes of stratification due to the unstable nature of village populations and unreliable estimates of village populations in Cambodia. Due to logistic reasons, the control households were not screened throughout the study period; and therefore were all, therefore, screening of all control households was conducted completed at the end of the study period.

### Sample size

All villages in Pailin were considered eligible to be included in this study. Preliminary analysis of microscopy data from the national malaria survey conducted in Cambodia in 2010 indicated that among households with at least one *Plasmodium* infection, 16 % had other individuals in the same household found to be *Plasmodium*-infected using microscopy. Therefore, it was estimated that with PCR, a more sensitive diagnostic tool, additional *Plasmodium*-infected individuals would be identified in 20 % of households with an index case. With a precision of 5, 95 % confidence interval and expected refusal rate of 10 %, it was estimated that 270 index cases should be followed up and included in the study to evaluate if 20 % of index households had at least one afebrile infected individual. The total number of households required with this expected proportion (p = 0.2; q = 1−0.2), a precision of 5 % (e), and a 95 % confidence interval (Z = 1.96) was N = (Z2pq)/e2~246 households +10 % refusal rate 270 index cases (households).

### Data collection

All members of selected households were invited to participate in the study. Each member was asked to provide a single finger-prick blood sample for preparation of multi-species RDT (SD Bioline Malaria Ag *P.f/*Pan and* P.f/P.v*), single thick and thin blood film, and three blood spots on filter paper for subsequent analysis by PCR. Index cases did not provide a blood sample at the household; only their original RDT diagnosis from the health centre or VMW was available. In addition, a structured case investigation form was completed by interviewing the head of each household, in order to collect information about demographic indicators and potential risk factors, including travel history, work history, and use of malaria prevention methods by the individual residents in the household. History of malaria infection was defined as having ever had malaria in the past. Net use was defined as having slept under a mosquito net (either standard or treated nets) the night before being interviewed. All responses to questions were self-reported.

### Laboratory methods

Thin blood films were fixed with methanol on the day of preparation by VMWs or health facility staff, and slides were stained with Giemsa according to standard practice at health facilities. Blood spots were dried and packaged into individual sealed plastic bags with desiccant. Samples were stored at 4 °C (in a standard refrigerator) up to 3 weeks after collection until they were transferred to the Institut Pasteur in Cambodia (IPC) for PCR analysis. Each blood spot was cut with a sterile 3 mm diameter hole-puncher and placed in a 96-well plate in numerical order. Samples were lysed overnight in a Saponin solution, and DNA was subsequently extracted using InstaGene Matrix resin, as previously described [31]. Samples were screened for the presence of *Plasmodium* DNA using a qualitative real-time PCR assay targeting *Plasmodium* cytochrome b gene. Positive samples were then analysed for *Plasmodium* species using four real-time PCR assays specifically amplifying *P. falciparum, P. vivax, Plasmodium ovale* and *Plasmodium malariae* [31].

### Statistical analyses

Paper-based questionnaires were anonymized and double-entered, along with all laboratory results, into an EpiData (EpiData Association, Odense, Denmark) template, which included range and type checks to minimise entry errors. PCR results were supplied by IPC in Microsoft Excel format, and merged with the core data using a unique ID code. However, due to variations in quality of slides prepared by local health staff and missing slides, it was decided to exclude all microscopy data from the analysis.

Data were analysed in STATA software, version 12.0 (StataCorp, College Station, TX, USA). Index case demographics and risk factors were compared to non-index cases to better understand what might lead to greater risk of infection. Infection rates and afebrile parasitaemia were compared between different household categories (index, neighbouring, and control). The proportion of screened household members with infection was also stratified by village incidence (comparing villages with <5 to villages with ≥5 malaria cases) and by febrile *versus* afebrile cases. Logistic regression models were developed to investigate individual level risk factors associated with RDT and PCR-identified infection. In addition, the proportion of additional individuals positive by PCR and RDT that would have been identified using screening approaches based on different risk factors (e.g. fever, use of nets, and history of malaria) was estimated.

### Variable selection

To explore the factors significantly associated with RDT- and PCR-identified infection, univariate logistic regression models were developed along with multivariate logistic regression models. These models used a backward step-wise approach, re-testing all included variables in the final minimal model. Predictors found to be borderline significant p value <0.1 were kept in the multivariate model. Models allowed for within-household correlation. Separate models were prepared with *Plasmodium* positivity by species as the primary outcome for both RDT and PCR diagnostic tests. However, due to the poor fit of the RDT models resulting from low case numbers, both species were combined for a multivariate RDT model.

The variables included in the model presence of fever, household category, distance to forest, village burden based on 2013 Malaria Information System data, history of malaria infection, being an adult male, and net use. Presence of fever was defined as having a temperature >37.2 °C using a digital axillary thermometre. Household categories included index households, the five closest households to index cases, and the ten closest households to index cases, as well as high incidence and low incidence control households. Distance to forest was categorized as <500 m, 500 m to 1 km, 1–2 km, and 2–5 km. Village burden was categorized as those greater than or less than five cases in 2013.

### Ethics

This study was approved by the Cambodian National Ethics Committee for Health Research (0064 NECHR). Blood safety was ensured by collecting samples using sterile techniques. VMWs were already fully trained in blood collection methods, but additional training was given on specific RACD procedures before the study took place. Signed consent was requested from the household head and individuals or their guardians (in the case of children) before taking blood samples, administering questionnaires, and any other form of data collection. Individuals who had positive RDTs were provided with treatment according to the Cambodian National Treatment Guidelines.

## Results

### Demographic description

Of 270 passively detected index cases diagnosed with RDT, the great majority (91 %) had *P. vivax* infection (Table 1). PCR was not conducted on index cases. 96 % of all households were contacted within 3 days of notification (the other 4 % were not contacted at all and therefore excluded from the analysis). It was not possible to match 15.9 % of samples taken for PCR to the data set, so these were also excluded from analysis.

**Table 1:** Plasmodium infection among index cases, individuals from non-index and control households, diagnosed by RDT and by PCR

	Index cases (passive detection)	Non-index individuals (active detection)	Comparison individuals
N tested by RDT	270	1898	183
n positive by RDT (%)	270 (100)	9 (0.5)	0 (0.0)
n *P. falciparum* (% of all RDT positive)	21 (7.8)	2 (22.2)	0
n *P. vivax* (% of all RDT positive)	245 (90.7)	7 (77.8)	0
n mixed by RDT (%)	4 (1.5)	0 (0.0)	0
N tested by PCR	n/a	1596	237
n positive by PCR (%)	n/a	17 (1.1)	25 (10.5)
n *P. falciparum* by PCR (%)	n/a	0 (0.0)	25 (100)
n *P. vivax* by PCR (%)	n/a	15 (88.0)	0 (0.0)
n mixed by PCR (%)	n/a	2 (12.0)	0 (0.0)

### Infections identified by RACD

Out of 1898 people screened by RDT using RACD, nine were positive (0.5 %). Seven (78 %) had *P. vivax* infections and two (22 %) had *P. falciparum* infections. Seven (78 %) infections were afebrile (Table 1). Out of 1596 people tested by PCR using RACD, 17 (1.1 %) were positive; with 15 being *P. vivax* infections (88 %) and two (12 %) being *P. falciparum/P. vivax*-mixed infections. Of the 16 PCR-positive individuals who had their temperature taken 14 (88 %) were afebrile. The proportion of individuals testing positive in index households (1.3 %), and the five (0.5 %) or 10 (0.9 %) nearest households was similar between all categories (Table 2).

**Table 2:** Plasmodium infection, stratified by household screening approach, including breakdown of afebrile infections

	RDT			PCR		
	N tested	n positive (%)	N afebrile (% of positives)	N tested	n positive (%)	N afebrile (% of positives)^a^
Index household	1266	4 (0.3)	3 (75.0)	1047	13 (1.3)	11 (91.7)
Five households closest to index household	236	3 (1.3)	2 (66.7)	200	1 (0.5)	0 (0)
Ten households closest to index household	396	2 (0.5)	2 (100)	349	3 (0.9)	3 (100)
Total in RACD households	1898	9 (0.5)	7 (77.8)	1596	17 (1.1)	14 (87.5)
Randomly selected households from high incidence villages	78	0 (0)	n/a	118	19 (16.1)	12 (75.0)
Randomly selected households from low incidence villages	105	0 (0)	n/a	119	6 (5.0)	3 (60.0)
Total in randomly selected control households	183	0 (0)	n/a	237	25 (10.5)	15 (71.4)

^a^Temperature data missing for some individuals,  % calculation according to correct denominators

### Infections identified in randomly selected comparator households

None of the 183 people screened by RDT in control households were positive. Out of 237 people from control households tested by PCR, 25 (10.5 %) were positive with all infections being *P. falciparum* (16 % in the high risk villages and 5 % in the low risk villages). While the majority of infections from households identified through RACD were *P. vivax*, all PCR positive infections in the control households were *P. falciparum*. From those who had their temperature taken (21/25) 71 % were afebrile.

### Predictors of *Plasmodium* spp. infection in a pre-elimination setting

Risk factors found to be a significant predictor for being RDT positive were fever (OR = 8.37, 95 % CI 1.54-45.59, p < 0.01), and history of malaria infection (OR 8.31, 95 % CI 1.95–35.40, p < 0.01). Mosquito net use was not included in the model since all RDT positive individuals slept under a net the night before. Risk factors found to be significant predictors for being *P. falciparum* positive by PCR were: presence of fever (OR 3.94, 95 % CI 1.33–11.65, p < 0.01); living in a randomly selected high incidence control household (OR 148.17, 95 % CI 28.92–759.01, p < 0.01); living in a randomly selected low incidence control household (OR 35.43, 95 % CI 7.02–179.03, p < 0.01); history of malaria infection (OR 6.62, 95 % CI 1.08–40.46, p = 0.04); and sleeping under a mosquito net the night before (OR 0.19, 95 % CI 0.52–0.68, p = 0.01). Interestingly, sleeping under a net was associated with protective effect despite the usage of nets being almost universal (96 %). The main predictor *P. vivax* infection by PCR was history of malaria infection (OR 12.11, 95 % CI 4.48–32.75, p < 0.01), and a borderline statistical significance was found with fever (OR 4.03, 95 % CI 0.95–16.90, p = 0.06), (Table 3).

**Table 3:** Univariate associations between factors investigated for association with *Plasmodium* infection by RDT and by PCR, and adjusted odds ratios, 95 % confidence intervals and p values for variables retained in final multivariate models

	Univariate			Multivariate		
Variable	Crude OR	95 % CI	p	Adjusted OR	95 % CI	p
*P. falciparum* positive by PCR^a^						
Household category						
Index household	1.00	–	–	1	–	–
Five closest HH	1.00	–	–	1	–	–
Ten closest HH	1.00	–	–	1	–	–
High incidence control HH	100.37	23.06, 436.79	<0.001	148.17	28.92, 759.01	<0.001
Low incidence control HH	27.77	5.83, 132.17	<0.001	35.43	7.02, 179.03	<0.001
Village burden 2013						
<5 cases in 2013	1.00	–	–	–	–	–
≥5 cases in 2013	1.74	0.70, 4.34	0.231	–	–	–
Village distance to forest						
<500 m	1.00	–	–	–	–	–
500 m–1 km	1.62	0.58, 4.58	0.360	–	–	–
1–2 km	0.55	0.07, 4.44	0.576	–	–	–
2–5 km	1.43	0.36, 5.71	0.609	–	–	–
Measured fever (>37.2 °C)	11.92	4.31, 32.91	<0.001	3.94	1.33, 11.65	0.013
Adult male (vs. all other age/sex groups)	1.58	0.80, 3.12	0.184	–	–	–
Used mosquito net last night	0.30	0.90, 0.97	0.045	0.19	0.52, 0.68	0.011
Previous malaria (ever)	1.58	0.40, 6.21	0.512	6.62	1.08, 40.46	0.041
*P. vivax* positive by PCR^a^						
Household category						
Index household	1.00	–	–	–	–	–
Five closest HH	0.40	0.05, 3.18	0.387	–	–	–
Ten closest HH	0.69	0.14, 3.37	0.647	–	–	–
High incidence control HH	1.00	–	–	–	–	–
Low incidence control HH	1.00	–	–	–	–	–
Village burden 2013						
<5 cases in 2013	1.00	–	–	–	–	–
≥5 cases in 2013	2.85	0.60, 13.58	0.188	–	–	–
Village distance to forest						
<500 m	1.00	–	–	–	–	–
500 m–1 km	0.60	0.13, 2.83	0.519	–	–	–
1–2 km	0.55	0.07, 4.26	0.568	–	–	–
2–5 km	0.35	0.04, 2.82	0.327	–	–	–
Measured fever (>37.2 °C)	4.54	1.06, 19.41	0.041	4.03	0.95, 16.90	0.058
Adult male (vs. all other age/sex groups)	1.60	0.72, 3.58	0.245	–	–	–
Used mosquito net last night	0.62	0.08, 4.63	0.641	–	–	–
Previous malaria (ever)	4.47	1.05, 19.11	<0.001	12.11	4.48, 32.75	<0.001
All species, positive by RDT						
Household category						
Index household	1.00	–	–	–	–	–
Five closest HH	4.08	0.99, 16.89	0.052	–	–	–
Ten closest HH	1.67	0.19. 14.47	0.642	–	–	–
High incidence control HH	–	–	–	–	–	–
Low incidence control HH	–	–	–	–	–	–
Village burden 2013						
<5 cases in 2013	1.00	–	–	–	–	–
≥5 cases in 2013	1.03	0.25, 4.31	0.964			
Village distance to forest						
<500 m	1.00	–	–	–	–	–
500 m–1 km	3.56	0.80, 15.80	0.095			
1–2 km	1.00	–	–	–	–	–
2–5 km	1.337466	0.14, 12.41	0.798	–	–	–
Measured fever (>37.2 °C)	9.36	1.74, 50.31	0.009	8.37	1.54, 45.59	0.014
Adult male (vs. all other age/sex groups)	2.902508	0.90, 9.35	0.074	–	–	–
Used mosquito net last night	–	–	–	–	–	–
Previous malaria (ever)	8.71	2.05, 37.05	0.003	8.31	1.95, 35.40	0.004

^a^ Mixed infections considered as positive for each species

### Consequence of limiting screening to certain criterion

If fever had been the only criterion for screening in this study, only 22 % of both RDT- and PCR-detected infections in the population would have been identified, with only 4 % of individuals requiring screening. Adding non-use of bed nets to the criterion would have identified two additional *P. falciparum* PCR-detected cases, or 27 % of all infections, and no additional RDT-detected cases. Only 7 % of individuals would have required screening. Adding history of malaria as a criterion would have identified five additional *P. vivax* infections, one additional *P. falciparum* infection, and one additional *P. falciparum*/*P. vivax mixed* PCR-detected case (40 % of total PCR infections), as well as two additional RDT-detected infections (44 % of RDT-sensitive infections), with 21.7 % of individuals requiring screening.

## Discussion

*Plasmodium* parasitaemia in households of index cases detected by PCD in this study was extremely low (0.5 % by RDT and 1.1 % by PCR), suggesting that screening of index households with RDT or PCR may not reveal as many additional cases in this setting as originally hypothesized. There was little difference between the reservoirs identified in index households versus neighbouring households, suggesting that targeting an RACD strategy to index households alone is insufficient to identify all infections in this setting. This is inconsistent with other studies using similar RACD approaches; nevertheless in those studies the difference in positivity between the household categories was small (1–2 %) and the number of cases quite low (23–74) [10, 13].

Fever was found to be a main risk factor associated with *Plasmodium* parasitaemia. In fact, many RACD strategies in other settings already use fever as a screening tool [10]. Absence of mosquito nets and a history of prior malaria were also associated with infection. Targeting individuals with those risk factors only for RACD screening would have reduced the number of individuals screened by 78 %, but identified only 40 % of all infections, and therefore is not justified in a setting targeting elimination. This contrasts with a study in Zambia, where limiting RDT screening to neighbours residing within the index case compound and neighbours with recent travel or fever could have identified 87 % of cases, while screening 79 % fewer individuals [10]. This suggests that RACD approaches need to be tailored to the specific geographical location where they are being implemented, depending on the level of transmission and the importance of specific risk factors.

It is important to note, however, that all control household infections were *P. falciparum* infections, while the majority of index and surrounding household’s infections were *P. vivax* infections. This result was not consistent with another RACD study in Zambia, where much higher infection rates were found in index (8 %) than control households (1 %) [12]. Control households in the current study were sampled at the end of the data collection period; their high infection rate could be explained by a small *P. falciparum* outbreak, which may have started after completion of other RACD screening. However, government surveillance data does not show any *P. falciparum* cases in the months before, during, or after control households were sampled (Malaria Information System data, 2014). Data from the 2013 Cambodia Malaria Survey found that although 28.7 % individuals with fever sought treatment at public facilities (including VMWs), 58.3 % went to private providers, and 12.9 % did not seek treatment [32]. It is possible that infected individuals instead sought treatment from private providers, which do not routinely submit case data to the government surveillance system, and therefore were not included in our RACD index case definition.

During the study it was not possible to collect any samples for PCR analysis from index cases. As a result, it was not possible to conduct molecular analysis and genotyping of identified *Plasmodium* parasites, which would allow us to determine if infections within the index households were similar to, and thus likely transmitted by, the index case. This study also relied on VMWs or health facility staff to collect blood samples from households; consequently the blood slides were of varying quality. Similar limitations may be faced when implementing RACD in other settings and special efforts to improve training and supervision should be taken to ensure high quality of slides.

Recent research suggests that sub-microscopic infections may not substantially contribute to the infectious reservoir [14]. These low-density infections, however, are still considered potentially infectious [34]. The usefulness of RACD in Pailin and its potential impact, if scaled up, is influenced by the extent to which very low-density infections contribute to on-going transmission within a community. If low-density infections are found to make up a large part of the infectious reservoir, screening target populations may be more cost-effective than focusing on periodic treatment follow-up (to identify treatment failures) and enhancing early detection in other ways. It is suggested that elimination programmes should consider how sensitive a diagnostic test is needed to identify all of those who comprise the infectious reservoir, as well as the likely spatial and temporal stability of transmission hotspots when designing any RACD approach [14].

Another limiting factor for RACD is the location of exposure. If the majority of exposure to infection occurs outside the community, then screening people according to residence is not appropriate. The results of this study strongly suggest that there is limited local transmission around the household, since very few secondary infections were identified. Therefore in this setting, RACD does not effectively target those most likely to have afebrile parasitaemia; however it may be more appropriate in settings with slightly higher transmission and different vector species. In order to better determine the incidence threshold at which RACD successfully identifies secondary infections, it is recommended that similar studies be conducted across a range of transmission settings.

In addition to questions about the effectiveness of RACD’s effectiveness for that specific setting, logistical feasibility must also be considered. For example, it was found that a team of 5–6 people employing this approach could visit two households per day and screen up to six household members. The time it takes to reach households may be significantly increased if RACD is adopted on a larger scale and integrated into the national programme’s routine activities, as was shown in Swaziland [13]. The level of experience of field staff should also be considered, particularly if microscopy is included; it was challenging to ensure a high quality of slides prepared by health centre staff. It is recommended that significant effort be invested in training staff in slide preparation. If PCR is required, shipping samples to laboratories that can provide molecular testing delays diagnosis [24]. In Cambodia, mobile PCR laboratories have been created to generate PCR results within 24 h; expanding this strategy could prove to be extremely resource intensive [31]. Balancing timeliness and sensitivity of diagnostic tools in RACD will need to be considered by countries when exploring RACD design and implementation.

Recent research shows that Mass Screening and Treatment (MSAT) may be more effective than individual screen and treat strategies, suggesting MSAT may be needed to successfully treat the afebrile parasite reservoir and reduce transmission [15, 25, 35]. At present, there is limited evidence showing the effectiveness of MSAT as a malaria elimination tool. Two pilot studies in Cambodia in 2008 and 2009 found that MSAT was not possible due to a lack of human resources [24]. Thus, it has since been suggested that targeted mass treatment (TMT), which does not require individual diagnostic results and is therefore faster than FSAT/MSAT, should be undertaken. It is hypothesised that TMT would address the challenge of infections with low parasitaemia which are not reliably detected by standard PCR methods. However, similar issues shared by RACD strategies around defining coverage and reaching the most at-risk populations may exist. In addition to operational challenges, implementation of TMT has to balance the risk of an increase in drug pressure on the parasite population, with the advantage of accelerating malaria elimination before resistance worsens [36, 37].

In most settings, introducing a new case investigation and response methodology requires a fundamental shift in the way surveillance is carried out at peripheral level. Given the limited evidence available, number of different options available, and due to the varying epidemiology between countries, it is unlikely that there will be a “one size fits all” solution. If elimination programmes find that RACD integrates well with their strategic plan, a careful evaluation of necessary methods, targeting, population coverage, interventions, and impact is in order [15].

There may also be a need to combine different approaches, or apply different strategies in different transmission settings within the country. Researchers are also working to improve data that can be used to drive these approaches. One example is the use of high spatial and temporal resolution surveillance data to identify hotspots [38, 39]. Combining better data sources with appropriate, evidence-based approaches for response can help guide us to possible malaria elimination in the near future [40].

### Study limitations

All PCR analyses conducted were subject to quality assurance procedures by qualified and experienced laboratory technicians, including positive and negative controls for both the extraction and PCR stages, and any invalid run was repeated in full. It has been shown that PCR analysis of small blood volumes (e.g. 5 µL) eluted from dried blood spots may miss some low density infections which are identifiable with PCR on larger volume venous blood samples (1 mL) [33], however, it is not thought that the unexpected findings in this study could be solely attributed to errors or limitations of PCR.

## Conclusion

This study found that RACD identified very few secondary infections when targeting screening to index and neighbouring households within a community. It is recommended that RACD is not appropriate for very low transmission settings where exposure to malaria occurs away from the community (e.g. in forested areas), and there is a high level of treatment-seeking from the private sector. RACD could be adapted to include index cases from the private sector, but is most appropriate in settings with declining and heterogeneous transmission, settled and stable populations, and where the primary vector habitat is within communities.
